# Case series of neuroretinitis in Korea

**DOI:** 10.1186/s12886-024-03290-3

**Published:** 2024-01-18

**Authors:** Seung Kwon Choi, Ik Soo Byon, Han Jo Kwon, Sung Who Park

**Affiliations:** 1 Department of Ophthalmology, Pusan National University College of Medicine, Busan, South Korea; 2 Department of Ophthalmology, Biomedical Research Institute, Pusan National University Hospital, 179, Gudeok-ro, Seo-gu, Busan, 49241 South Korea

**Keywords:** Neuroretinitis, Ocular toxocariasis, Optic neuritis, Toxocara, Toxocariasis

## Abstract

**Background:**

To present the clinical characteristics of neuroretinitis in Korea.

**Methods:**

Twelve patients with neuroretinitis between January 2009 and September 2020 were retrospectively reviewed. Neuroretinitis was diagnosed based on fundus findings, optical coherence tomography, and fluorescein angiography. The serological findings of each patient were reviewed.

**Results:**

Fifteen eyes of 12 patients (9 male and 3 female), with a mean age of 46.0 ± 10.7 years were included. Of the nine patients who underwent serological testing for Toxocara antibodies, six (66.6%) were positive. One patient had high titers of Toxoplasma immunoglobulins M and G. One patient diagnosed with dengue fever was suspected to have neuroretinitis in both eyes. There were no related abnormalities in the serological findings in four patients (33.3%) out of 12 patients. There were no suspected cases of cat-scratch disease. The six patients who were positive for Toxocara antibodies were older (mean age: 54.5 ± 9.1 years) than the others (mean age: 37.5 ± 4.4 years, *p* = 0.004). The four patients without any abnormal serological findings were relatively younger (mean age: 35.7 ± 3.0 years) than the other 8 patients (mean age: 51.1 ± 10.1 years, *p* = 0.008).

**Conclusions:**

Two-thirds of neuroretinitis patients were seropositive for Toxocara in the current cohort from Koreans. Causative factors in cases of neuroretinitis may vary according to age and region.

## Introduction

Neuroretinitis is an inflammatory disease characterized by optic disc edema and macular exudates. Its nomenclature has varied and includes stellate retinopathy, neuroretinitis, Leber’s idiopathic stellate neuroretinitis, or optic disc edema with a macular star [1]. In 1916, Leber first reported a patient with acute unilateral visual loss with disc edema and macular exudates, naming the condition “stellate maculopathy” [2]. In 1977, Gass employed fluorescein angiography (FA) to demonstrate that the site of leakage was not the macula, but the optic disc, and thus suggested the term “neuroretinitis”, which was later confirmed by reports using advanced imaging modalities [3–5].

The pathogenesis however remains unclear to date. Various infectious organisms or infectious conditions have been suspected to have relation with neuroretinitis, including syphilis [6, 7], Lyme disease [8], tuberculosis [9], herpes simplex [10], toxoplasmosis [11, 12], toxocariasis [13, 14], nematodes [15], and dengue fever [16]. Suhler et al. [17] reported that nine out of 14 patients with neuroretinitis had antibodies against *Bartonella Henselae* in the USA. Kahloun et al. [18] reported that the their patients were related with cat-scratch disease (30.8%), rickettsiosis (19.2%), and idiopathic neuroretinitis (23.1%) in North Africa.

To the best of our knowledge, there has been no case series of neuroretinitis in Asians. We are to report 12 cases of neuroretinitis from Korea.

## Materials and methods

We conducted a retrospective study of patients who were diagnosed with neuroretinitis between January 2009 and September 2020 at Pusan National University Hospital. Initial cohort of patients was identified using embedded searching system based on diagnostic code. Seventeen patients were included. Three patients were excluded given that they were suspected to have other etiologies such as retinal vasculitis, hypertensive retinopathy and hematologic disorder. Two patients were excluded because they did not perform examination enough to set up the diagnosis.

The current study was approved by the Institutional Review Board of Pusan National University Hospital (2011-020-097) and was conducted in accordance with the principles of the Helsinki Declaration. Neuroretinitis was diagnosed based on clinical symptoms, fundus examination, optical coherence tomography (OCT), and FA.

History taking, visual acuity examination, slit-lamp examination, fundus examination, OCT, and FA were performed, as were additional visual field and magnetic resonance imaging (MRI) examinations according to the treating physician’s decision. Laboratory work-up was not performed in all patients, as Toxocara or Toxoplasma antibody tests were not always available and tests for various infectious diseases with very low incidence were conducted only when indicated. However, in a majority of patients, tests for specific infectious causes including syphilis, toxoplasmosis, and toxocariasis, and others including complete blood cell counts, blood chemistry, and chest radiography, were conducted.

In all patients with a clinical diagnosis of neuroretinitis, optic disc edema was confirmed and FA revealed distinct leakage around optic disc in the early phase and macular area pooling in the late phase (except Case 12). The cases with no other abnormalities related to inflammation (retinitis, choroiditis or scleritis) were included. Exudative maculopathy was defined as presence of stellate macular exudates on fundus photography or subretinal fluid within one disc diameter from the center of the macula on OCT.

Categorical variables collected were analyzed using Fisher’s exact test. The Mann-Whitney test was used to analyze the continuous variables, including best-corrected visual acuity (BCVA), which was converted to logMAR units for statistical analysis. Data analysis was performed using SPSS for Windows (version 22.0; SPSS Inc., Il, USA).

## Results

A total of 15 eyes of 12 patients with neuroretinitis (10 eyes of 9 male patients and 5 eyes of 3 female patients) were included. For 3 of the 12 patients, both eyes were involved. The mean age was 46.0 ± 10.7 (33–69) years. The chief complaints were decreased visual acuity in 9 patients, glare in one, metamorphopsia in one, and visual field impairment in one patient. Four patients exhibited a viral prodrome. The clinical and demographic data of the patients are summarized in Table 1.

**Table 1:** Patient clinical and demographic data

Patient No.	Sex	Age	Eye	Influenza-like symptoms	Presumed causatives	Duration of symptom (days)	Initial Visual Acuity*	Final Visual acuity*
1	M	48	OD	(-)	Toxocara	5	20/20	20/20
			OS	(-)	Toxocara		20/40	20/25
2	M	55	OD	(+)	Toxocara	1	20/60	20/25
3	M	69	OS	(-)	Toxocara	5	20/800	20/400
4	M	34	OD	(+)	idiopathic	2	20/50	20/25
5	M	33	OS	(-)	idiopathic	2	20/200	20/20
6	F	36	OD	(-)	idiopathic	3	20/25	20/20
7	F	40	OD	(-)	idiopathic	1	20/20	20/20
			OS	(-)			20/20	20/20
8	M	45	OD	(-)	Toxoplasma	3	20/2000	20/2000
9	M	42	OS	(-)	Toxocara	30	20/50	20/40
10	M	55	OS	(-)	Toxocara	14	20/20	20/20
11	M	58	OD	(+)	Toxocara	14	20/40	20/25
12	F	37	OD	(+)	Dengue fever	2	20/800	20/30
			OS	(+)			20/800	20/30

M = male, F = Female, * Snellen visual acuity

The initial mean logMAR BCVA was 0.74 ± 0.83 (0–1.7) and the mean central macular thickness was 401.2 ± 136.3 μm (223–638). Six of the 9 patients (66.6%) showed positive for Toxocara antibodies. These six patients took oral albendazole (800 mg/day) for two weeks. The Toxoplasma antibody test was done in 10 of the 12 patients, and it was revealed that one (Case 8) had a high titer for IgG (> 300 IU/ml) and IgM (0.5 IU/ml) of Toxoplasma antibody. Case 12, with a history of traveling to Thailand, had antibodies against the dengue virus. There were no related abnormalities in the laboratory findings of four patients (Case 4, 5, 6, 7), and their visual acuity was relatively preserved during their clinical course even without any special treatment (Table 2).

**Table 2:** Serologic test results

Patient No.	Toxocara IgG	Toxoplasma IgM	Toxoplasma IgG	Rapid Plasma Reagin	Dengue fever	Bartonella
1	Positive	Negative	Negative	Negative	NA	NA
2	Positive	Negative	Negative	Negative	NA	NA
3	Positive	Negative	Negative	Negative	NA	NA
4	Negative	Negative	Negative	Negative	NA	NA
5	NA	NA	NA	Negative	NA	NA
6	Negative	Negative	Negative	Negative	NA	NA
7	Negative	Negative	Negative	Negative	Negative	Negative
8	NA	Positive	Positive	Negative	NA	NA
9	Positive	Negative	Negative	Negative	NA	NA
10	Positive	Negative	Negative	Negative	NA	NA
11	Positive	Negative	Positive	Negative	NA	NA
12	NA	NA	NA	Negative	Positive	Negative

NA: non-available

The six patients positive for Toxocara antibodies were older than the others (54.5 ± 9.1 years versus 35.7 ± 3.0, *p* = 0.008). In contrast, the four patients (Case 4, 5, 6, 7) without any specific serological findings were younger than the others (35.7 ± 3.0 versus 51.1 ± 10.1, *p* = 0.008). The final visual acuity was 20/400 in the case 3 who was with positive for Toxocara antibody and in an immunocompromised state. The final visual acuity was 20/2000 in the case 8 who was suspected to be ocular toxoplasmosis. Other than the case 3 and 8, the visual acuity of thirteen eyes recovered to 20/63 or higher.

This cohort includes four etiologies; Toxocara, Toxoplasma, Dengue virus, and idiopathic. A representative case of each etiology is described below.

### Case 11

A 58-year-old man presented with decreased visual acuity in the right eye that went on for 2 weeks. He drank liquor made with hand-picked wild fruit 2 weeks prior to his initial visit. He had experienced headaches the day after drinking the liquor, and developed a central scotoma in the right eye 2 days following the onset of the headaches. The BCVA was 20/40 in the right eye and 20/20 in the left eye.

**Fig. 1:**
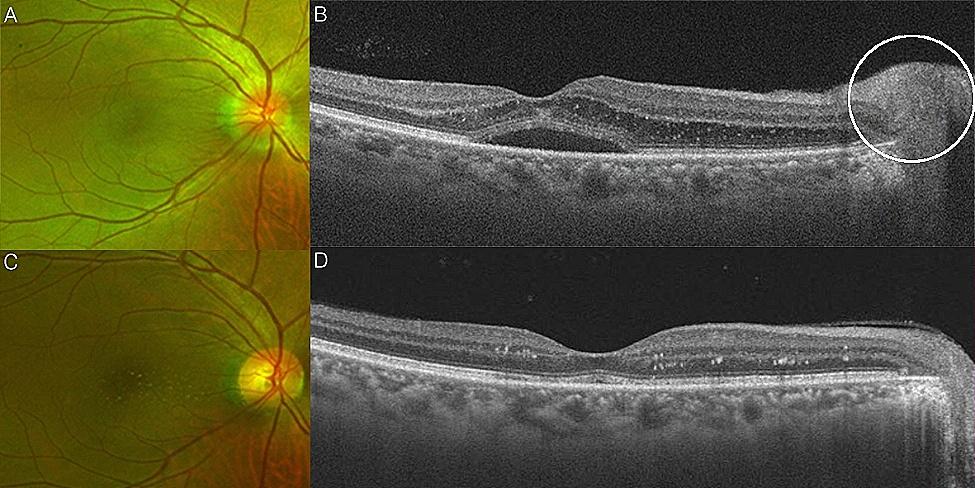
Clinical manifestation of a 58-year-old man (Case 11) with neuroretinitis. Fundus photo showed optic disc edema of the right eye during his initial visit (**A**), optical coherence tomography of the right eye showed subretinal fluids (**B**), and optic disc edema (white circle). 1 month after albendazole treatment, optic disc edema disappeared and hard exudates were clearly noticed in the fundus photo (**C**). In the optical coherence tomography, subretinal fluid was nearly absent and hard exudates became more distinct (**D**)

Fundus photography (Fig. 1A) and OCT (Fig. 1B) showed optic disc edema and subretinal fluid in the right eye. Laboratory test results were positive for Toxocara antibodies. Oral albendazole (1200 mg/day) was prescribed for 7 days. After 1 month, the subretinal fluid disappeared on OCT (Fig. 1D), and optic disc edema improved on fundus photography (Fig. 1C). Stellate macular deposits were observed on fundus photography and OCT (Fig. 1C and D).

### Case 8

**Fig. 2:**
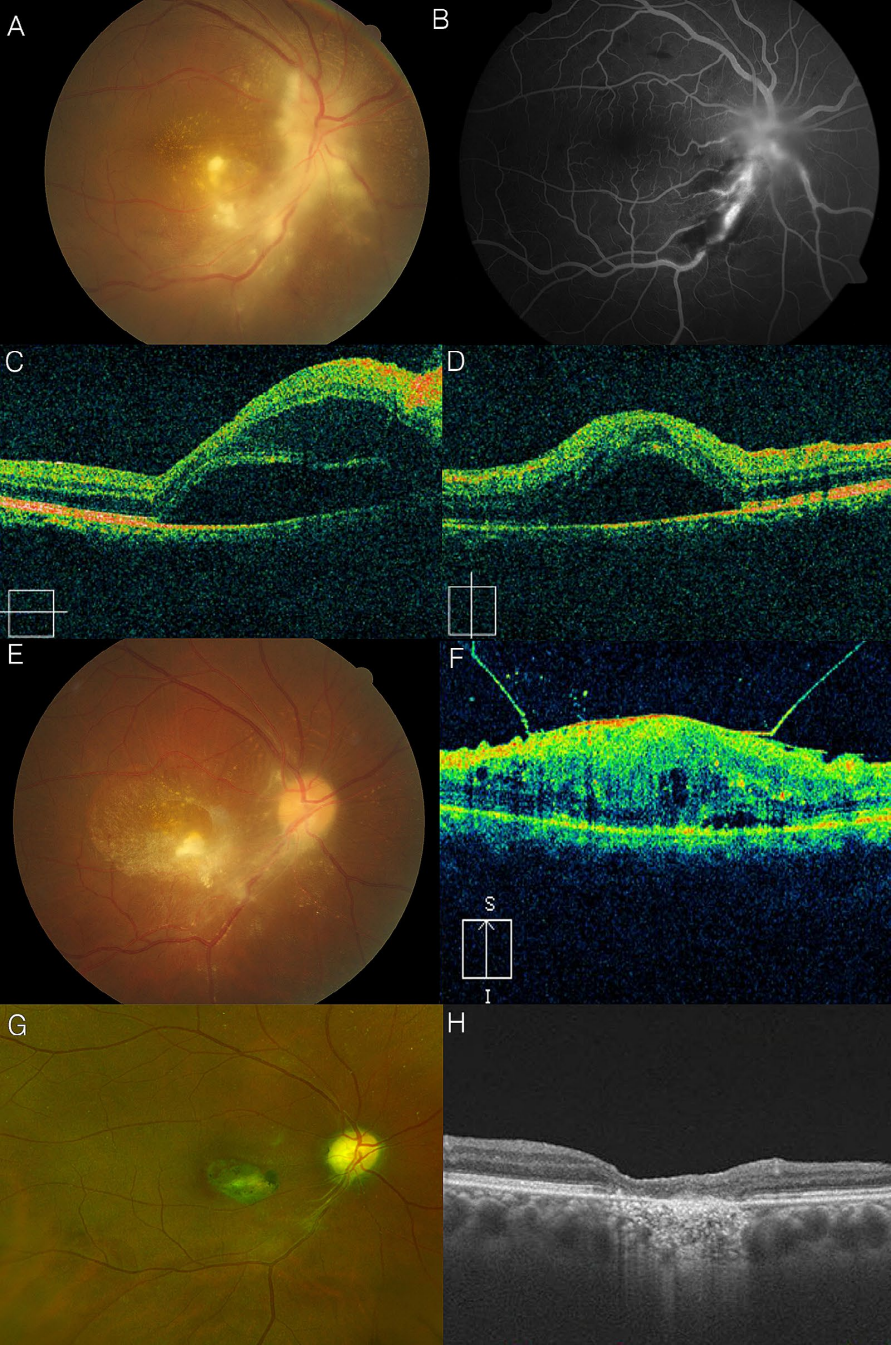
Clinical manifestation of a 45-year-old man (Case 8) with high titers of Toxoplasma immunoglobulins G and M. Fundus photo showed multifocal edematous and opaque retina and optic disc edema in his right eye (**A**), and fluorescein angiography showed that inflammatory focus was around the optic disc. (**B**). Subretinal fluid was observed, which continued into the optic disc head in optical coherence tomography (**C**, **D**). At 4 months after anti-Toxoplasma treatment, stellate macular exudates and several tractional membranes were observed in the fundus photo (**E**) and in optical coherence tomography (**F**). At 6 years after epiretinal membrane removal, chorioretinal scarring on the macula and pale optic disc were observed (**G**, **H**). The best-corrected visual acuity was finger count

A 45-year-old male patient presented with sudden onset vision loss in his right eye 3 days prior to his initial visit. The symptoms began after a day of heavy drinking. BCVA was counting fingers, and multiple areas of opaque retina were observed on fundus photography (Fig. 2A). Peripapillary subretinal fluid was observed on OCT (Fig. 2C and D) and FA (Fig. 2B) demonstrated optic disc leakage. Laboratory tests showed positive results for Toxoplasma antibodies (IgG > 300 IU/mL, IgM 0.55). Oral trimethoprim-sulfamethoxazole, and clindamycin were prescribed for 2 months. After 3 months, optic disc edema and subretinal fluid in the right eye improved, but severe epiretinal membranes were observed (Fig. 2E and F). Vitrectomy with membrane peeling was performed. Six years later, a chorioretinal scar on the macula were observed along with optic nerve pallor (Fig. 2G and H). BCVA was counting fingers at this time.

### Case 12

A 37-year-old woman was hospitalized secondary to a fever of unknown origin, which started while traveling in Thailand. She had fever, diarrhea, nausea, and vomiting. After returning to Korea (3 days after the onset of fever), laboratory investigations and empirical oral antibiotics, including doxycycline and ciprofloxacin, were prescribed. There were no specific findings in the laboratory tests other than a mild neutropenia (1231 cells/µL). Following empirical treatment, her general condition improved. At that time, she complained of visual discomfort in both eyes for the first time, which began with fever though. She was subsequently referred to the Department of Ophthalmology.

**Fig. 3:**
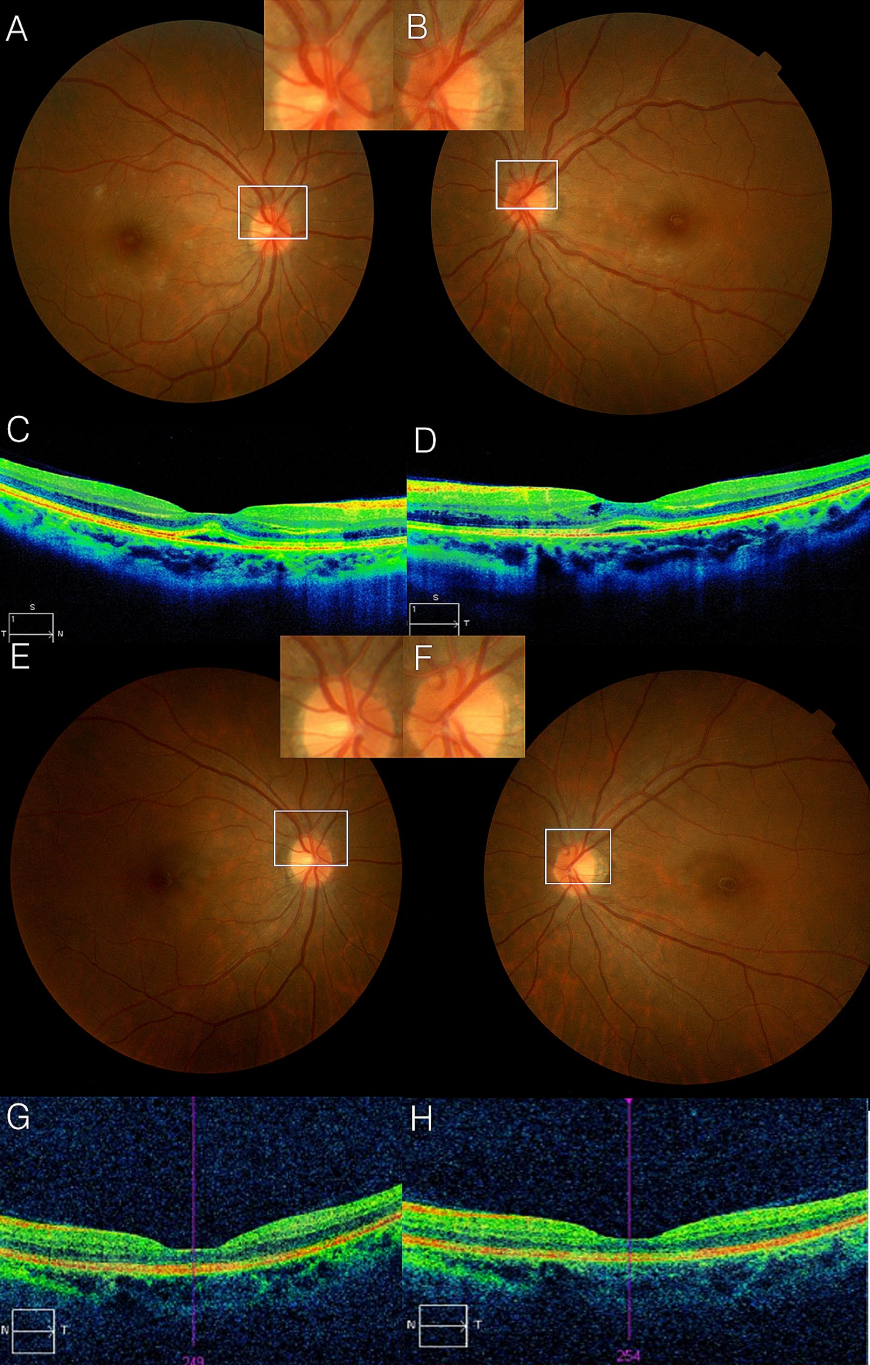
Clinical manifestation of a 37-year-old woman (case 12) with dengue fever. At 7 days after empirical antibiotics therapy for the fever, fundus photography (**A**, **B**) revealed the slightly blurred margin of the optic nerve head (white boxes), which was suspected to be in the process of recovering from optic disc swelling. Optical coherence tomography showed scant subretinal fluids (**C**, **D**). At 1 month after the initial visit, fundus photos (**E** and **F**) and optical coherence tomography (**G**, **H**) showed that these abnormalities returned to normal

Figure 3A and B show fundus photos and OCTs on the 7th day after prescribing empirical antibiotics. The BCVAs were 20/63 of the right eye and 20/50 of the left eye, respectively. The OCTs showed a subretinal fluid. The margin of the optic head appeared slightly blurred and was suspected to be in the process of recovering from optic disc swelling. The patient did not want any further ophthalmic examinations. Considering the travel history of the patient to Thailand, further serological tests were conducted, and she was found to be positive for antibody to Zika virus (Dengue fever) and negative for antibody for *Bartonella Henselae* (Cat scratch disease). Thus, a diagnosis of dengue fever was made.

Fundus photographs (Fig. 3C and D) and OCTs (Fig. 3C, and D) showed mild optic nerve edema, and subretinal fluid which resolved 1 month following her first ophthalmic visit. Compared to the previous ones (Fig. 3A and B), the optic disc appeared slightly pale (Fig. 3C and D) on follow up. Her BCVA recovered to 20/25 in both eyes. Optic disc edema and subretinal fluid was eventually met with complete resolution (Fig. 3E, F, G, and H).

### Case 7

**Fig. 4:**
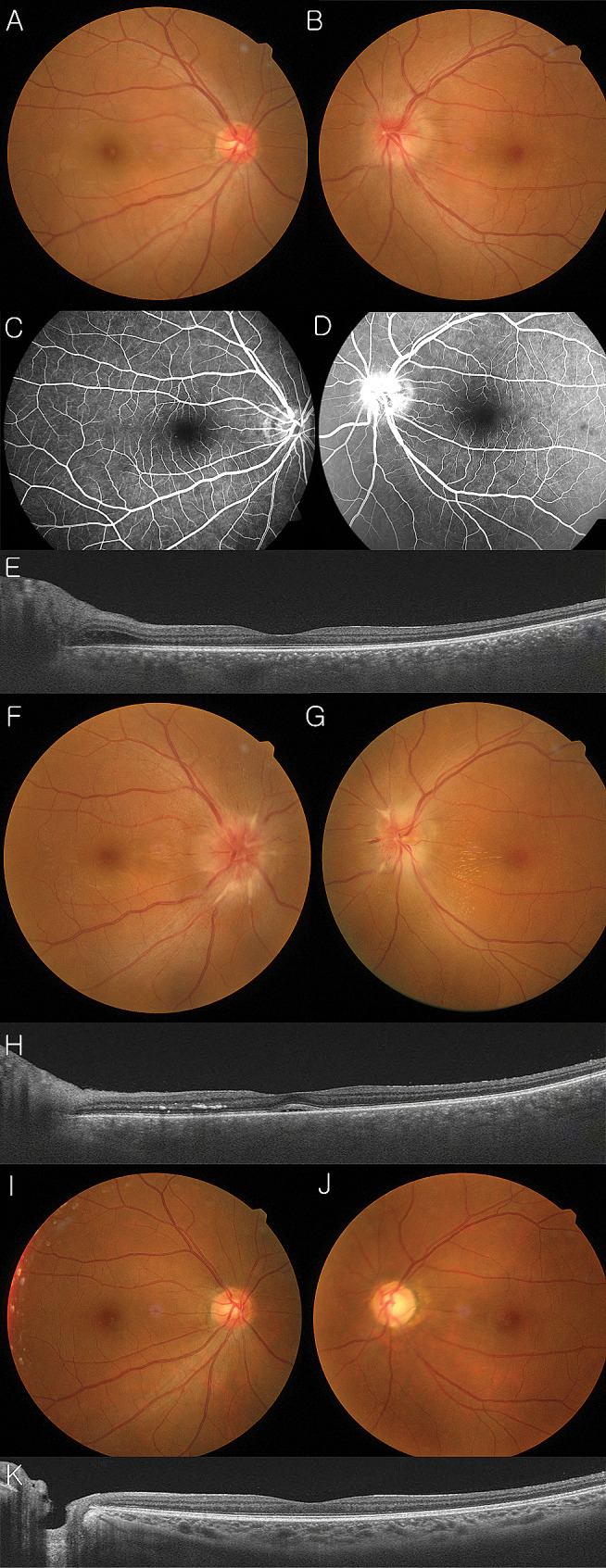
Clinical manifestation of a 40-year-old woman without any specific laboratory findings. Fundus photographs showed optic disc edema of only the left eye (**A**, **B**), optic disc swelling was observed, although there was no macular abnormality seen in optical coherent tomography (**E**) at the initial visit. Fluorescein angiography (**C**, **D**) showed that the inflammatory focus was around the optic disc of the left eye. Three weeks after the initial visit, optic disc swelling was noticed in both eyes (**F**, **G**), and subretinal fluid and hard exudates were observed in optical coherent tomography of her left eye (**H**). Three months later, without any specific treatment, these abnormalities returned to almost-normal (**I**, **J**, **K**)

A 40-year-old woman presented with a sudden onset of central scotoma that she had experienced in her left eye 1 day prior to the initial visit. She denied a viral prodrome. No specific medical history was noted. BCVA was 20/20 in both eyes. Fundus photography (Fig. 4A) and OCT (Fig. 4C) showed optic disc edema and peripapillary retinal edema in the left eye. Her right eye did not demonstrate any abnormal findings (Fig. 4B and E). FA revealed peripapillary leakage of the left eye (Fig. 4D). Laboratory investigations were normal. As optic neuritis was suspected, she was referred to a neurologist and underwent CSF analysis and MRI, which were normal. The lesions were closely observed without medication.

Four weeks later, subretinal fluid of the left eye and optic disc edema of the right eye were newly observed (Fig. 4F, G, and H). BCVA decreased to 20/25 in the right eye and 20/30 in the left eye. She was diagnosed with neuroretinitis in both eyes. Serological tests, including those to rule out infectious etiologies such as cat-scratch disease, were negative. Seven weeks later, even without any treatment such as steroid or antibiotics, optic disc edema and macular edema resolved (Fig. 4I, J, and K), and BCVA recovered to 20/20 in both eyes.

## Discussion

Neuroretinitis is a rare disease that typically presents with abrupt visual loss, optic disc edema, and lipid exudates in a macular star appearance [19]. In two previous studies [17, 18], the average age of incidence was 30 years of age, with equal prevalence between men and women. Unilateral eye involvement was common [17, 18], though several bilateral cases have also been reported [11, 12]. In the case series by Suhler et al. [17], more than half of the patients had symptoms of a viral prodrome preceding the development of neuroretinitis.

In 1916, Leber first reported disease with optic disc edema and macular exudates of the eye along with a sudden decrease in visual acuity, naming the condition “idiopathic stellate maculopathy” [2]. Gass, who named it “neuroretinitis” for the first time, illuminated via FA that the macular star originates from leakage secondary to optic disc edema, not primarily from the retina [20]. Kitamei et al. [5] identified lipid-containing fluid leakage from a single arteriole in the superficial nerve fiber layer of the optic disc. These results suggest a central role in peripapillary and optic nerve inflammation in the pathophysiology of neuroretinitis, which may lead to the characteristic macular exudates seen in this condition. Fluid is absorbed through the retinal pigment epithelium, and the lipid components of the fluids take the form of stellate macular exudates [20].

Neuroretinitis was reported to be caused by various infectious agents, including syphilis [6, 7, 21], toxoplasmosis [11, 12], toxocariasis [13, 14], tuberculosis [9], Lyme disease [8], and leptospirosis [19]. Suhler et al. [17] reported that two-thirds of their analyzed neuroretinitis cases in the USA (9 of 14) were associated with *Bartonella Henselae* (cat-scratch disease). In a Tunisian study, eight out of 26 patients with neuroretinitis were positive for cat-scratch disease [18].

*Bartonella Henselae* is the most common cause of neuroretinitis in previous reports [17–19]. However, none of the patients in the current cohort had specific symptoms related to cat-scratch disease; the serological tests of two patients (cases 7 and 12) were negative for this disease. As cat-scratch disease is extremely rare in Korea [22], the laboratory test for *Bartonella Henselae* has low accessibility in Korea; blood samples needed to be sent to a specific center (Korea Centers for Disease Control and Prevention), and it took more than 10 days to obtain the results. This is the reason why the laboratory test was performed only in cases 7 and 12 in this study.

Humans can be infected by ingestion of embryonated eggs of Toxocara from contaminated sources, such as soil, undercooked meat, and fresh or unwashed vegetables. Toxocariasis is one of the main infectious sources of uveitis in Korea [23]. Koreans, especially elderly males, regularly ingest raw meat, including raw cow liver, which is thought to be the main route of Toxocara infection in Korea.

Toxocara invades the retina, optic nerve, or brain [14, 24, 25]. In particular, two reports [24, 25] showed that the optic nerve may be a route in which Toxocara parasites infect the retina. A few cases of Toxocara-related neuroretinitis have been reported, most of whom were Koreans^12,24^. The Toxocara antibody test was performed in nine out of 12 patients in the current cohort, and six of them showed positive results. These six patients were all male and over 40 years of age.

There is no consensus for the treatment for ocular toxocariasis. In the current study, five of the six patients (Except case 7) who were positive for Toxocara were treated with albendazole 1200 mg/day for 7 days without any steroids. Their final visual outcomes were relatively good (LogMAR 0.098 ± 0.10, equal to 20/25). One patient (Case 7) who was immunocompromised secondary to terminal cancer was not treated with anti-helminthic medication or steroids. This patient presented with poor initial BCVA (20/400) and was receiving chemotherapy to treat his rectal cancer. Bevacizumab (0.05 cc) was injected intravitreally once, but it was ineffective.

Dengue fever is a disease caused by an arbovirus transmitted by the *Aedes aegypti* and *A. albopictus* mosquito, which is endemic to Southeast Asia and Southeast Africa [26]. Dengue fever rarely affects the eye. To the best of our knowledge, a few cases involving the posterior segment of the eyes have been reported. One of them was bilateral neuroretinitis in 2006 [16] and neuroretinitis resolved in only a few day after treatment with antibiotics. The case 12 was diagnosed with Dengue fever, however, was unclear that neuroretinitis was combined. It is because the initial ophthalmic examinations were only conducted 7 days after antibiotic treatment, disease activity was not distinctive in the fundus photos and OCTs. In addition, the patient refused further ophthalmic evaluations, including FA. The series of fundus photographs and OCT in Case 12 were not sufficient to confirm neuroretinitis. However, because the OCT signal represented lipid deposits and fundus photographs showed blurred margins of the optic nerve head, it was assumed that the disease was neuroretinitis, which was being improved by antibiotics.

Laboratory evaluations were negative in four (33.3%) out of 12 cases. Kahloun et al. [18] reported that 6 (23%) of 26 patients were idiopathic in their cohort. Purvin et al. [27] assumed a viral etiology for all idiopathic cases, but this has yet to be proven. Purvin et al.^27^ had reviewed all reported cases at that time and summarized that idiopathic neuroretinitis usually affected young adults, with more than half experiencing a preceding flu-like illness. Most of such patients recovered with excellent visual outcomes with conservative management. The current data accorded with the previous data, except for flu-like symptoms. Four (33%) of 12 patients in our cohort that were classified as idiopathic were younger than the others, and although they had not been treated with any systemic medication, their visual outcomes were excellent. Only one idiopathic patient (Case 4) experienced flu-like symptoms.

There were several limitations in our study, including its retrospective nature and small sample size. The laboratory investigations were dependent on each treating physician and therefore were not uniform. In particular, tests for *Bartonella Henselae* were performed in only two cases. The images of Case 12 were not sufficient to diagnose her with neuroretinitis.

To the best of our knowledge, this is the first case series of neuroretinitis in Asia. Unlike previous reports conducted in other regions, there were no cases related to cat-scratch disease, and toxocariasis was suspected to be the most common cause.

In summary, the causative factors of neuroretinitis can vary according to patient age and region of residence. Toxocariasis is presumed to be the most common cause of neuroretinitis in Korea. Anti-helminthic drugs were prescribed without steroids in cases positive for Toxocara, which was met with good visual outcomes.

## Data Availability

All data generated or analyzed during this study are included in this published article.
